# *Flammulina filiformis* *Pkac* Gene Complementing in *Neurospora crassa* Mutant Reveals Its Function in Mycelial Growth and Abiotic Stress Response

**DOI:** 10.3390/life12091336

**Published:** 2022-08-28

**Authors:** Yayong Yang, Bin Xie, Zhuohan Jing, Yuanping Lu, Jun Ye, Yizhao Chen, Fang Liu, Shaojie Li, Baogui Xie, Yongxin Tao

**Affiliations:** 1 College of Horticulture, Fujian Agriculture and Forestry University, Fuzhou 350002, China; 2 Mycological Research Center, College of Life Sciences, Fujian Agriculture and Forestry University, Fuzhou 350002, China; 3 State Key Laboratory of Mycology, Institute of Microbiology, Chinese Academy of Sciences, Beijing 100101, China

**Keywords:** filamentous fungi, cAMP-dependent protein kinase, heat and oxidative stresses, allogenic gene complement

## Abstract

*Flammulina filiformis* is a popular edible mushroom that easily suffers from heat and oxidative stresses. The cyclic adenylate-dependent protein kinase A (cAMP/PKA) pathway is the main signaling pathway in response to environmental stress, and the PKAC is the terminal catalytic subunit of this pathway. In this study, the *Pkac* gene was identified in *F. filiformis*, which was highly conserved in basidiomycetes and ascomycetes. The transcription analysis showed that the *Pkac* gene was involved in the mycelial growth and the fruiting body development of fungi. In *Neurospora crassa*, the *Pkac* gene deletion (ΔPkac) resulted in the slower growth of the mycelia. We complemented the *F. filiformis FfPkac* to *N. crassa* ΔPkac mutant to obtain the CPkac strain. The mycelial growth in the CPkac strain was restored to the same level as the WT strain. In addition, the *FfPkac* gene showed significantly up-regulated expression under heat and oxidative stresses. By analyzing the differentially expressed genes of ΔPkac and Cpkac with WT, respectively, seven downstream genes regulated by *Pkac* were identified and may be related to mycelial growth. They were mainly focused on microbial metabolism in diverse environments, mitochondrial biogenesis, protein translation and nucleocytoplasmic transport. RT-qPCR results confirmed that the expression patterns of these seven genes were consistent with *FfPkac* under heat and oxidative stresses. The results revealed the conserved functions of PKAC in filamentous fungi and its regulatory mechanism in response to heat and oxidative stresses.

## 1. Introduction

The cyclic adenylate-dependent protein kinase A (cAMP/PKA) signal pathway is known as a major signal transduction pathway in eukaryotes in response to extracellular signal stimulation and plays an important role in regulating the growth and development of an organism [1,2]. Adenylate cyclase (AC) and PKA are two major catalytic enzymes in the cAMP/PKA signal pathway. AC is activated by the active G protein α subunit and catalyzes ATP to form the second messenger cyclic adenylate (cAMP) [1,3,4,5]. PKA is composed of two PKA catalytic subunits (PKAc) and two PKA regulatory subunits (PKAr) [6,7]. The cAMP binds to two PKAr when its concentration reaches a certain level, resulting in conformational changes of PKA tetramer R2C2. The two PKAc subunits are released, and phosphorylate target substrates, including metabolic enzymes and transcription factors [6,7,8]. As the main effector in the last step of the cAMP signaling pathway, *PKAc* gene deletion or mutation is helpful in identifying a series of target genes downstream of the cAMP/PKA pathway.

In fungi, cAMP/PKA signal transduction is reported to be involved in the regulation of fungal mycelial growth, morphogenesis, pathogenicity, etc. [2,5,9,10]. The *PKAc* gene was also performed by gene deletion or overexpression to understand its effect on fungal life. In *Pleurotus ostreatus*, the overexpression of *PKAc* could significantly improve the transcription levels of the genes encoding lignin-modifying enzymes, indicating that *PKAc* plays an important role in inducing wood degradation and mycelial nutritional growth [11]. In contrast, when the *PKAc* gene was deleted, the radial growth of mycelia was absent in *Fusarium verticillioides* [12], or the transition from budding to filamentous growth was disrupted in *Ustilago maydis* [13]. It suggests that the *PKAc* gene plays important regulatory roles in the vegetative growth of filamentous fungi. Whether this function of the *PKAc* gene is conserved in filamentous fungi needs further direct experimental verification. Moreover, *PKAc* gene deletion also affected the infection efficiency of the *F. verticillioides* [12,14], the production of melanin or virulence of *Cryptococcus neoformans* [15], indicating the possible role of the *PKAc* gene in regulating metabolism and responding to the environment.

In the life history of filamentous fungi, mycelial growth, primordia formation and fruiting body development are regulated by environmental factors such as temperature and light. Moderate environmental stress and oxidative stress are the key inducers of morphogenesis of mycelia and fruiting bodies [16]. *Flammulina filiformis* is a popular edible mushroom and a typical agaric fungus, which belongs to basidiomycete. Its fruiting body is suitable to grow at low temperatures and can be cultivated in industrialized style in a cold mushroom room. *F. filiformis* easily suffers from many environmental stresses such as heat and oxidative stresses in the production process. Therefore, exploring the function of the cAMP/PKA signal pathway in response to stress is important for the cultivation and production of *F. filiformis*. As another model filamentous fungus, *Neurospora crassa* belongs to ascomycete and is easy to operate in genetics. More than 70% of genes in the *N. crassa* genome have corresponding knockout mutants, and it is an ideal material for studying the function of the conserved genes in fungi. Thus, the *Pkac* gene was identified in *F. filiformis* and *N. crassa* first in this study. The expression of *Pkac* in response to abiotic stresses was analyzed in *F. filiformis*. Meanwhile, the *Pkac* gene in *F. filiformis* was complemented into the *Pkac* knockout mutant of *N. crassa* (ΔPkac). The possible downstream genes regulated by the *Pkac* gene were also identified and compared to the *Pkac* knockout mutant and complementary stain (CPkac) in *N. crassa*. The results will be helpful for understanding the conserved function of *Pkac* in filamentous fungi and its regulatory mechanism of the cAMP/PKA signal transduction pathway.

## 2. Materials and Methods

### 2.1. Strains and Culture Conditions

*F. filiformis* dikaryotic strain 1123, obtained by mating monokaryotic strains L11 and W23, was provided by the Fujian Edible Fungi Germplasm Resource Collection Center of China. *N. crassa* wild type strain FGSC#4200 (WT), the *NcPkac* (gene No. NCU00682) knockout mutant strain FGSC#11433 (ΔPkac) and plasmid pCB1532 were provided by the Institute of Microbiology, Chinese Academy of Sciences. The *Escherichia coli* DH5α was used for vector construction, purchased from Tiangen BioTech Co., Ltd. (Beijing, China). The *F. filiformis* strains and *N. crassa* strains were maintained on potato dextrose agar (PDA) medium and Vogel’s medium [17] at 25 °C, respectively.

Cultivation of the fruiting body of strain 1123 was performed as per the method described by Tao et al. [18]. The fruiting body was sampled at six different developmental stages after inoculation, including mycelia (MY), primordia (PR), pileus in elongation stage (ELP), stipe in elongation stage (ELS), pileus in maturation stage (MAP), and stipe in maturation stage (MAS).

For the collection of mycelia, *F. filiformis* strain 1123, L11 and W23 were cultured on the PDA medium with the cellophane sticking firmly. When the mycelia were fully grown, they were treated with heat stress (HS) at 37 °C or oxidative stress (H_2_O_2_) at 5, 10 and 20 mM for 1 h, respectively. The samples were collected and stored at −80 °C.

To compare the growth rate, the equipotent hypha blocks with mycelia of WT, ΔPkac and CPkac strains of *N. crassa* were inoculated onto a new 90 mm Vogel’s medium plate and incubated in the dark at 25 °C for 12 h. The length of mycelial growth was measured with a vernier caliper, and the growth rate of the colony was calculated. The viable monokaryotic microconidia were purified from conidiating strains of *N. crassa* grown on Westergaard and Mitchell synthetic crossing (SC) medium supplemented with iodoacetate (IAA) via the method of Ebbole et al. [19].

### 2.2. Sequence, Structure and Similarity Analysis of Pkac Gene in F. filiformis and N. crassa

According to the *NcPkac* gene in *N. crassa* (NCU00682), the orthologous gene *FfPkac* in the genome of *F. filiformis* L11 (GenBank accession number: APIA00000000.1; BioProject: PRJNA191865) was identified by local BLASTp searching. The sequence of *FfPkac* was then retrieved from the genome of the *F. filiformis* L11 strain. Open reading frame (ORF) of the *Pkac* gene was predicted by ORF Finder online software (http://www.ncbi.nlm.nih.gov/gorf/gorf.html, accessed on 25 June 2022). The structure of *Pkac* gene was visualized by Gene Structure Display Server (GSDS 2.0) (http://gsds.gao-lab.org/, accessed on 25 June 2022). The PKAC protein conserved domain was predicted by InterProScan online software (http://www.ebi.ac.uk/Tools/pfa/iprscan/, accessed on 25 June 2022). Phylogenetic tree construction was performed using the neighbor-joining method of MEGAX software [20], and the bootstrap value was set to 1000. DNAMAN software (Lynnon Corporation, Vaudreuil-Dorion, QC, Canada) was used to conduct the multiple sequence alignment with full-length sequences of six PKAC proteins from *F. filiformis*, *N. crassa*, *Agaricus bisporus*, *Coprinopsis cinerea*, *Trichoderma reesei*, and *Cordyceps militaris*.

### 2.3. Total DNA and RNA Extraction and RT-qPCR

Total DNA was isolated from the samples of *F. filiformis* and *N. crassa* using a modified cetyltrimethylammonium bromide (CTAB) method [21]. Total RNA was isolated using an Omega E.Z.N.A. Plant RNA Kit (Omega Bio-Tek, Norcross, GA, USA) according to the manufacturer’s protocol. Extracted RNA was quantified using a NanoND-1000 spectrophotometer (NanoDrop Technologies, Wilmington, DE, USA). Only RNA samples with A260/A280 ratios between 1.9 and 2.1 were used for cDNA synthesis. The cDNA was synthesized using 1000 ng RNA for each sample, according to the instructions of a TransScript^®^ All-in-One First-Strand cDNA Synthesis SuperMix for qPCR (One-Step gDNA Removal) kit (Transgen, Beijing, China).

Real-time quantitative PCR (RT-qPCR) was performed using a CFX96 Real-Time PCR Detection System (Bio-Rad, Hercules, CA, USA). RT-qPCR amplification included a denaturation step of 30 s at 94 °C, followed by 35 cycles of 5 s at 94 °C and 30 s at 60 °C, according to the instructions of TransStart^®^ Top Green qPCR SuperMix (Transgen, Beijing, China). *ACTB*, *Ras* and *GAPDH* were used as internal control genes for the normalization of the RT-qPCR in this study [22]. All primers for RT-qPCR were designed by Primer Premier 5.0 software and are shown in Table S1 (Supplementary Materials). The relative expression levels of the tested genes were determined according to the 2^−∆∆Ct^ method [23].

### 2.4. Construction of Complementation Vector of N. crassa

The *FfPkac* gene was amplified from the cDNA of *F. filiformis* L11 with the primers *FfPkac*-F and *FfPkac*-R. The 5′ end and 3′ end homologous arm fragments were amplified from the DNA of *N. crassa* WT strain with the primer pairs *NcPkac*5′-F-AscI and *NcPkac*5′-R and *NcPkac*3′-F-NotIHF and *NcPkac*3′-R-AscI, respectively. The TrpC terminator fragment was amplified with the primers TrpC-F and TrpC-R-SpeIHF. The above four fragments were introduced into the linearized plasmid PCB1532 by a seamless cloning kit (Transgen, Beijing, China) to obtain a new plasmid named as FfPkac-C. The recombinant plasmids were transferred into receptor cells DH5α, and the positive transformants were screened in LB plates containing ampicillin. The recombinant plasmid was cleaved with restriction endonuclease AscI and transferred into the ΔPkac strain via the electroporation method [24]. The positive transformants were screened using medium containing chlorimuron-ethyl. All the primers employed for plasmid construction are shown in Table S1 (Supplementary Materials).

### 2.5. RNA-Seq of N. crassa Transformants

RNA-Seq was performed using Hiseqx-ten, which was entrusted to Biomarker Technologies Co, LTD (Beijing, China). The clean data were analyzed with HISAT, StringTie and Ballgown software via the method of Pertea et al. [25]. The transcription levels of all genes were quantified by standardized FPKM values. The differentially expressed genes between the WT, ΔPkac and CPkac strains of *N. crassa* were screened out via the method of Thomas et al. [26]. The homologous genes of *N. crassa* were found in *F. filiformis* by local BLAST software. The amino acid sequences of the differentially expressed genes were uploaded to the Kyoto Encyclopedia of Genes and Genomes (KEGG) for pathway annotation to predict their functions.

## 3. Results

### 3.1. Structure and Protein Function of Pkac Gene

**Figure 1:**
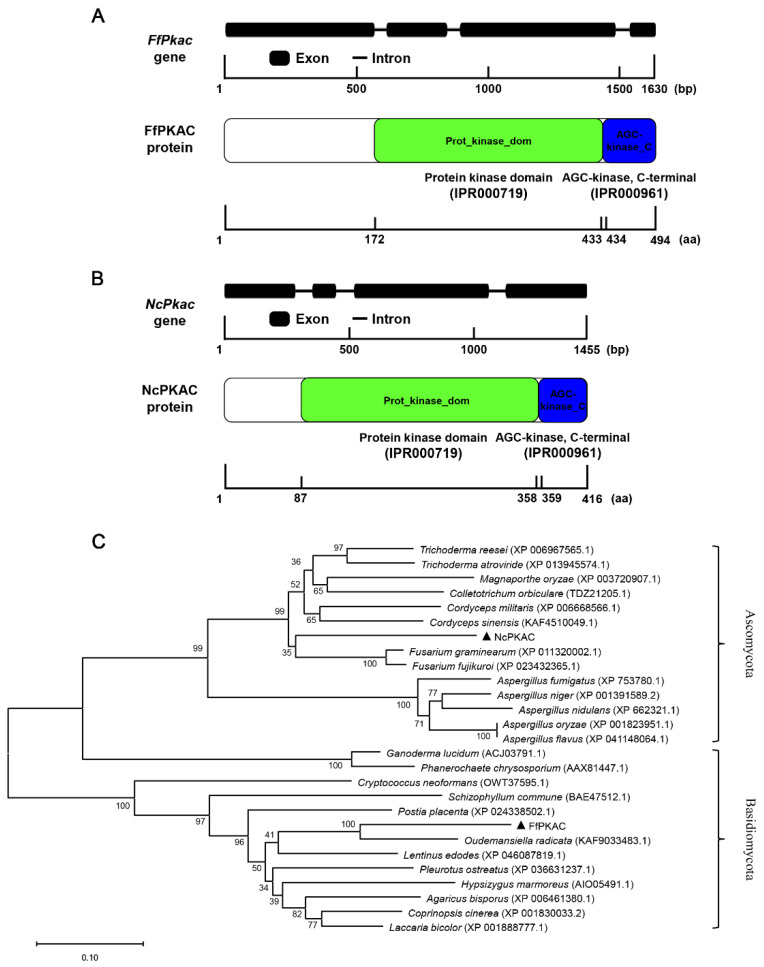
Gene and protein structures and phylogeny analysis of *FfPkac* and *NcPkac* in *Flammulina filiformis* and *Neurospora crassa*. (**A**) Gene structure of *FfPkac* and protein structure of FfPKAC in *F. filiformis*. Thick lines represent exons, and thin lines represent introns. The green rectangle and blue rectangle represent the protein kinase domain and AGC-kinase, C-terminal, respectively. (**B**) Gene structure of *NcPkac* and protein structure of NcPKAC in *N. crassa*. Thick lines represent exons, and thin lines represent introns. The green rectangle and blue rectangle represent the protein kinase domain and AGC-kinase, C-terminal, respectively. (**C**) Phylogeny analysis of PKAC. Black triangles represent FfPKAC in *F. filiformis* and NcPKAC in *N. crassa*, respectively. Phylogenetic tree of FfPKAC and NcPKAC proteins from other species, including basidiomycetes and ascomycetes.

The length of the *FfPkac* gene is 1630 bp, and its ORF encodes 494 amino acids, containing four exons and three introns (Figure 1A). The length of *NcPkac* gene is 1455 bp, its ORF encodes 416 amino acids, containing four exons and three introns (Figure 1B). Both FfPKAC and NcPKAC proteins contained two conserved domains: protein kinase domain (IPR000719) in the middle and AGC-kinase (IPR000961) in the C-terminal. The protein kinase domain is a conserved catalytic core and is important for the protein kinase activity for catalyzing protein phosphorylation. The AGC (cAMP-dependent, cGMP-dependent and protein kinase C) protein kinase domain has three conserved phosphorylation sites that serve as phosphorylation-regulated switches to control both intra-molecular and inter-molecular interactions and is critical for the catalytic activity (Figure 1A,B).

The evolutionary tree (Figure 1C) showed that FfPKAC and PKAC form *Ganoderma lucidum*, *Lentinus edodes*, *A. bisporus*, *P. ostreatus* and *C. cinerea* are clustered into basidiomycetes group; while NcPKAC and PKAC form *Fusarium graminearum*, *C. militaris*, *T. reesei* and *Aspergillus fumigatus* are clustered into the ascomycetes group. The evolution of the *Pkac* gene was consistent with the evolution of the species, suggesting the conserved function of the *Pkac* gene in fungi.

**Figure 2:**
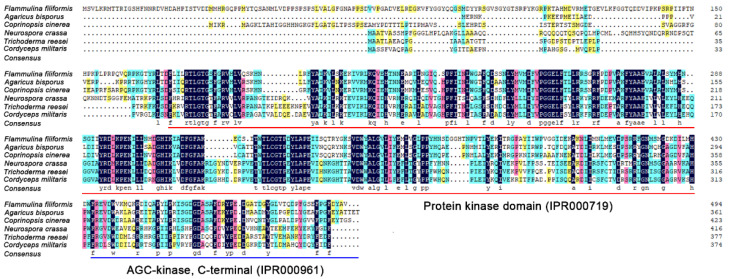
Multiple sequence alignment of PKAC protein sequences from different species.

PKAC of four species from basidiomycetes (*A. bisporus* and *C. cinerea*) and ascomycetes (*T. reesei* and *C. militaris*) were selected for multiple sequence alignment with *F. filiformis* FfPKAC and *N. crassa* NcPKAC. The result showed that these six PKAC proteins shared multiple conserved sites in two conserved protein kinase and AGC-kinase domains (Figure 2). It also suggested that PKAC may have highly conserved functions in filamentous fungi.

Red underline and blue underline represent protein kinase domain (IPR000719) and AGC-kinase, C-terminal (IPR000961), respectively.

### 3.2. Expression Pattern of Pkac Gene

**Figure 3:**
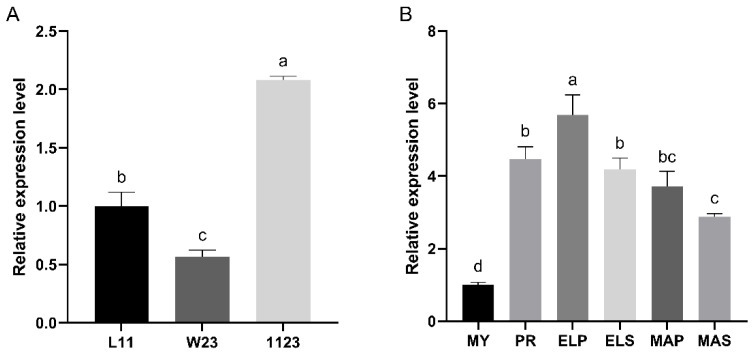
Relative expression levels of *FfPkac* in different strains and developmental stages. (**A**) The relative expression levels of *FfPkac* in three different strains (L11, W23, 1123) of *F. filiformis*. L11 and W23 are the monokaryotic strains and 1123 is the dikaryotic strain. (**B**) The relative expression levels of *FfPkac* at different developmental stages of strain 1123. MY (Mycelia) is the mycelia stage, PR (Primordia), ELP (Pileus in elongation stage), ELS (Stipe in elongation stage), MAP (Pileus in maturation stage), and MAS (Stipe in maturation stage) make up the fruiting body stage. (**A**,**B**) The values are the means ± SD of three independent experiments (Tukey’s multiple comparisons test: lowercase letters represent *p* < 0.05).

The relative expression levels of the *Ffpkac* gene in monokaryotic mycelia (L11 and W23), dikaryotic mycelia (1123) and fruiting bodies of *F. filiformis* at different developmental stages were detected by RT-qPCR (Figure 3). The relative expression level of *FfPkac* in dikaryotic mycelia was 2.1-fold and 3.7-fold higher than that in monokaryon L11 and W23, respectively (Figure 3A). The relative expression level of *FfPkac* was significantly up-regulated during the process of dikaryotic mycelia forming fruiting bodies in *F. filiformis*. Compared with that in MY stage, the transcription level of *FfPkac* was up-regulated 4.5-fold in the PR stage and maintained higher levels in ELP (5.7-fold), ELS (4.2-fold), MAP (3.7-fold) and MAS (2.9-fold) stages. It suggested that *FfPkac* may be related to the mycelial growth and development of the fruiting body of macro fungi.

### 3.3. Pkac Gene Regulates Mycelial Growth

**Figure 4:**
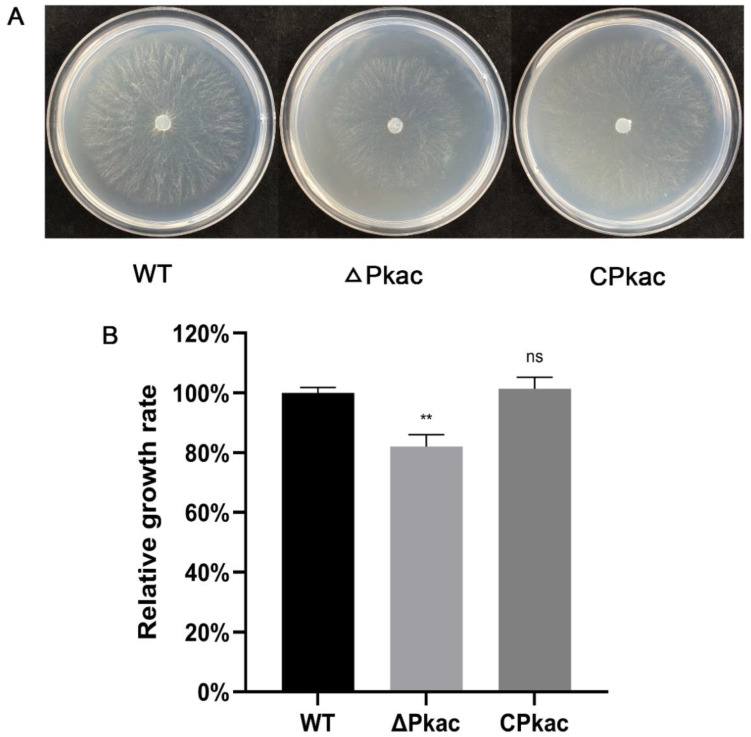
Mycelial growth rates of different strains of *N. crassa*. (**A**) Mycelial phenotype of *N. crassa* transformants grown on Vogel’s medium. (**B**) Relative growth rate of mycelia of *N. crassa* on Vogel’s medium for 12 h. The values are the means ± SD of three independent experiments. Asterisks indicate significant differences compared to WT (Dunnett T3′s multiple comparisons test: ** *p* < 0.01, ns: not significant).

As a model fungus, *N. crassa* has a rich gene mutant library including the *Pkac* knockout mutant. We firstly investigated the phenotype of the mycelial growth of the *Pkac* knockout mutant strain (ΔPkac), in which the *NcPkac* gene was replaced by the hygromycin gene. Compared with the WT, the mycelia of ΔPkac strain grew sparsely and slowly (decreased by 17.9%) (Figure 4A). In order to further confirm the conserved role of the *FfPkac* gene on mycelial growth, we complemented the *F. filiformis FfPkac* gene into the *N. crassa* ΔPkac strain and obtained the complemented strain of *N. crassa* named CPkac. Compared with the ΔPkac strain, the mycelia of the CPkac strain grew thicker and faster (Figure 4). The growth rate of mycelia in the CPkac strain was increased by 1.2-fold compared with ΔPkac and showed no significant difference to that in WT (Figure 4B). The results suggested that the *Pkac* gene played an essential role in mycelial growth and shared the conserved function in both *F. filiformis* and *N. crassa*.

### 3.4. Pkac Gene Participates in Abiotic Stress Response

**Figure 5:**
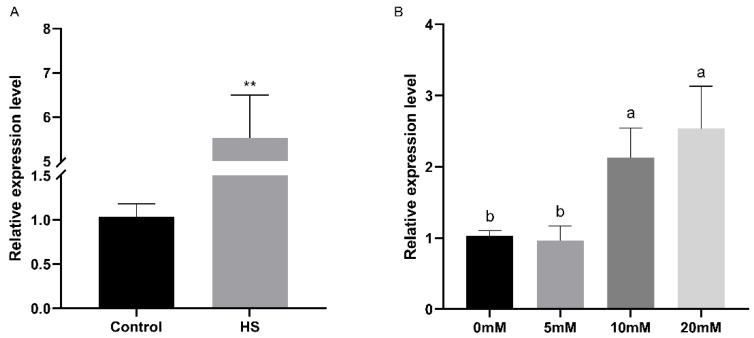
Relative expression levels of the *FfPkac* gene in *F. filiformis* under heat and oxidative stresses. (**A**) Relative expression levels of the *FfPkac* gene of *F. filiformis* in strain 1123 under heat stress. The values are the means ± SD of three independent experiments. Asterisks indicate significant differences compared to control (Student’s *t* test: ** *p* < 0.01). (**B**) Relative expression levels of the *FfPkac* gene of *F. filiformis* strain 1123 under different concentrations of H_2_O_2_. The values are the means ± SD of three independent experiments (Tukey’s multiple comparisons test: lowercase letters represent *p* < 0.05).

The relative expression levels of the *FfPkac* gene were detected under heat stress and at different concentrations of H_2_O_2_ stress in *F. filiformis* by RT-qPCR (Figure 5). The relative expression level of *FfPkac* was up-regulated 5.3-fold under heat stress (Figure 5A). Under the treatment of H_2_O_2_, the relative expression levels of the *FfPkac* gene showed a gradual upward trend with the increase in H_2_O_2_ concentration. Compared with that in the control (0 mM H_2_O_2_), the relative expression levels of the *FfPkac* gene were up-regulated 2.1-fold and 2.5-fold at 10 and 20 mM H_2_O_2_, respectively (Figure 5B). The relative expression levels of the *FfPkac* gene were significantly up-regulated under abiotic stress of heat and oxidative stresses, suggesting that the *FfPkac* gene played a role in coping with heat and oxidative stresses.

### 3.5. Identification and Validation of Downstream Regulatory Genes of Pkac

**Figure 6:**
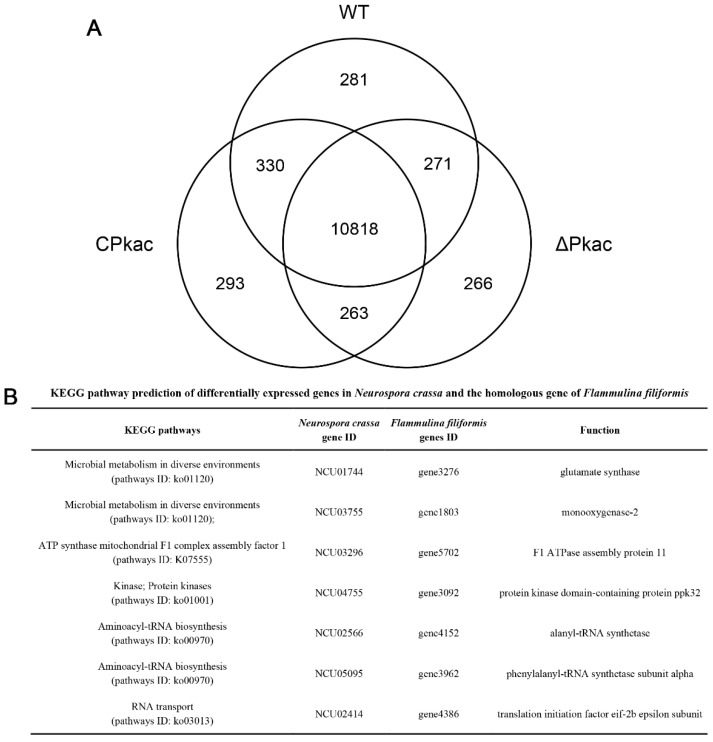
Analysis of differentially expressed genes in the transcriptome of *N. crassa*. (**A**) Wayne diagram of differentially expressed genes of *N. crassa*. (**B**) KEGG pathway prediction of differentially expressed genes in *N. crassa* and the homologous gene of *F. filiformis*.

In order to investigate the downstream acting genes of the *Pkac* gene, RNA-Seq was performed for three strains WT, ΔPkac and CPkac. There were 11,700, 11,618 and 11,704 expressed genes detected in WT, ΔPkac and CPkac, respectively. Among them, 10,818 genes were expressed in any of the three strains, accounting for 82.7% of the total genes. There were 281 (2.1%), 266 (2.0%) and 293 (2.2%) genes specifically expressed in WT, ΔPkac and CPkac strains, respectively, suggesting that these genes could be related to *Pkac* gene deletion and complementation (Figure 6A).

According to the FPKM values from RNA-Seq, three groups of genes, including (A) the differentially expressed genes between ΔPkac and WT, (B) the differentially expressed genes between ΔPkac and CPkac, and (C) the non-differentially expressed genes between WT and CPkac were screened, respectively. We took the intersection of A, B and C and obtained 38 genes in total after excluding the low expression genes (the average FPKM value < 20). These 38 genes underwent function annotation using the KEGG pathway and Pfam conserved domain analysis. Finally, there were seven genes with the defined functions: glutamate synthase (*N. crassa*: *NCU01744*/*F. filiformis*: *gene3276*); monooxygenase-2 (*N. crassa*: *NCU03755*/*F. filiformis*: *gene1803*); F1 ATPase assembly protein 11 (*N. crassa*: *NCU03296*/ *F. filiformis*: *gene5702*); protein kinase domain-containing protein ppk32 (*N. crassa*: *NCU04755*/*F. filiformis*: *gene3092*); alanyl-tRNA synthetase (*N. crassa*: *NCU02566*/*F. filiformis*: *gene4152*); phenylalanyl-tRNA synthetase subunit alpha (*N. crassa*: *NCU05095*/*F. filiformis*: *gene3962*); translation initiation factor eif-2b epsilon subunit (*N. crassa*: *NCU02414*/*F. filiformis*: *gene4386*). These seven genes belong to five KEGG pathways: Microbial metabolism in diverse environments (pathways ID: ko01120); ATP synthase mitochondrial F1 complex assembly factor 1 (pathways ID: K07555); Kinase; Protein kinases (pathways ID: ko01001); Aminoacyl-tRNA biosynthesis (pathways ID: ko00970); and RNA transport (pathways ID: ko03013) (Figure 6B). The functions and metabolic pathways of these seven genes were closely related to mycelial growth and response to stress, suggesting that *Pkac* may perform its functions by on acting these downstream genes.

**Figure 7:**
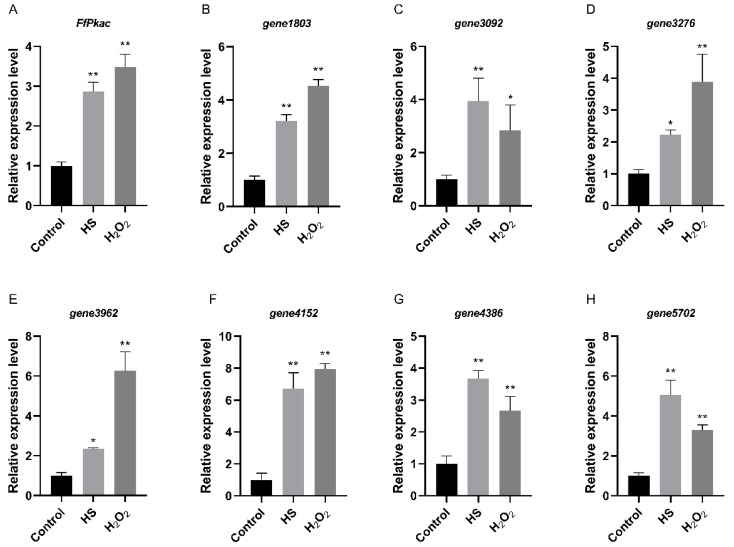
Relative expression levels between *FfPkac* and its downstream genes in *F. filiformis* under heat and oxidative stresses. (**A**) The relative expression levels of *FfPkac* in *F. filiformis* mycelia under heat and oxidative stresses. (Dunnett T3′s multiple comparisons test: ** *p* < 0.01). (**B**–**H**) The relative expression levels of seven downstream genes of *FfPkac* in *F. filiformis* mycelia under heat and oxidative stresses. (Dunnett T3′s multiple comparisons test: * *p* < 0.05, ** *p* < 0.01). In (**A**–**H**), the values are the means ± SD of three independent experiments. Asterisks indicate significant differences compared to control.

In order to verify the consistent expression patterns between *Pkac* and its downstream genes identified above, the expression levels of these seven genes, as well as *FfPkac,* were further detected by RT-qPCR under heat and oxidative stresses in *F. filiformis* (Figure 7). The results showed that the relative expression levels of the *FfPkac* gene were significantly up-regulated 2.9-fold and 3.5-fold under heat and oxidative stresses, respectively. These seven genes were up-regulated with different degrees under heat and oxidative stresses, respectively, and shared consistent expression patterns with *FfPkac*.

## 4. Discussion

The filamentous fungi are composed of many morphologically and developmentally diverse species, which can be divided into basidiomycetes and ascomycetes. For functional studies of many important conserved genes, it is necessary to compare and validate them in both basidiomycetes and ascomycetes. However, the ease of genetic manipulation and the efficiency of genetic transformation vary greatly among fungi, making it impossible to perform arbitrary gene function tests in most fungi, especially in basidiomycetes. *F. filiformis* and *N. crassa* can be used as a model species of basidiomycetes and ascomycetes, respectively, due to their rich phenotypes and profound research basis. In *N. crassa*, the genetics of operation is easy and efficient. More than 70% of the genes in the *N. crassa* genome have corresponding gene knockout mutants in its mutant library, while in most basidiomycetes, including *F. filiformis*, the genetic transformation efficiency is low, and to date, gene knockout operation is still a challenge. Therefore, it is a rapid approach to revealing the molecular function of many conserved genes in basidiomycetes by using the existing mutant library of the ascomycete *N. crassa* model. In this study, the gene *FfPkac* was successfully complemented into *N. crassa* ΔPkac mutant by site-specific repair mutagenesis technique and the mycelial growth phenotypic of ΔPkac strain in *N. crassa* was also restored to the same level as WT after the complementation of *FfPkac*. This proves the feasibility of the strategy of studying gene function by using mutants of the model fungus combined with gene complementation among different species.

The cAMP/PKA signal transduction pathway is one of the conservative signaling pathways in fungi. In this study, *FfPkac* (on behalf of the cAMP/PKA signaling pathway) was found to be involved in response to the heat and oxidative stresses in *F. filiformis*. In *N. crassa*, the transcription level of the *NcPkac* gene was found to up-regulate significantly after the addition of DTT (Dithiothreitol: induce Endoplasmic Reticulum stress) in WT and down-regulate after the addition of DTT in the mutant Δrrg-2 (*rrg-2* plays a role in the oxidative stress response based on the data from GSE61949 [27,28]). In addition, the cAMP/PKA signaling pathway is also found to be involved in response to heat shock and salt stress in *Saccharomyces cerevisiae* [29]. It suggests that the cAMP/PKA signaling pathway plays an essential role in response to a variety of environmental stresses universally. In edible fungi, the mycelial growth, the formation and development of fruiting bodies are affected and regulated by the external environment. Of course, the cAMP/PKA signaling pathway also plays a role in the growth and development of fungi. In this study, both the absence of *NcPkac* and the complementation of *FfPkac* significantly affected the mycelial growth. Based on the data GSE52153 of *N. crassa* [30], the transcription level of *NcPkac* in the Δczt-1 (CZT-1: cell death activated zinc cluster transcription factor) mutant strain (Δczt-1 was higher susceptible to the staurosporine compared with the WT) was significantly up-regulated compared to that in the WT. The roles of *Pkac* in the growth and development of fungi were also verified and validated in several species. In *Aspergillus niger*, the overexpression of *pkaC* could modify hyphal, colony and conidiophore growth [31]. In *F. verticillioides*, the *fpkl* gene (encoding a homolog of the PKA catalytic subunit) mutant showed reduced vegetative growth, fewer and shorter aerial mycelia, and the defects in radial growth and macroconidiation [12,14]. In *Botrytis cinerea*, growth and virulence were severely affected by deletion *bcpka1* [32]. In *Sporisorium scitamineum*, *PKA* is also involved in the proper mating and filamentation as well as virulence [10]. In addition, the sustained high expression with varying levels of *FfPkac* in different developmental stages of *F. filiformis* indicated that the cAMP/PKA signaling pathway is also essential for the fruiting body development.

The roles of PKA or the cAMP/PKA signaling pathway have been described in detail; however, there are few studies on the identification of downstream genes in this pathway. In this study, the possible downstream genes regulated by the *Pkac* gene were identified by comparing ΔPkac and CPkac with WT, respectively. The downstream genes of *Pkac* were mainly focused on microbial metabolism in diverse environments, mitochondrial biogenesis, protein translation, nucleocytoplasmic transport, etc. RT-qPCR confirmed that the expression patterns of these downstream genes were consistent with *FfPkac* in response to heat and oxidative stresses. In addition, ROS (reactive oxygen species) catabolic enzymes are also found to be under the regulation of the cAMP/PKA signaling pathway in *S. scitamineum* [10]. In the future, increased identification of PKAC downstream regulatory genes under different conditions will provide more powerful clues to fully reveal the regulatory mechanism of PKAC as well as the cAMP/PKA signaling pathway in fungi.

## Data Availability

All experimental data in this study will be made available upon reasonable request from readers.

## Supplementary Materials

The following supporting information can be downloaded at: https://www.mdpi.com/article/10.3390/life12091336/s1, Table S1: The primers used in this study.
