# Supplementary material for: Flammulina filiformis Pkac Gene Complementing in Neurospora crassa Mutant Reveals Its Function in Mycelial Growth and Abiotic Stress Response
Source: Life (Basel). 2022 Aug 28;12(9):1336. doi: 10.3390/life12091336 (PMC9502917; doi:10.3390/life12091336)
Supplement: Supplementary file 1 [file life-12-01336-s001.zip › life-1878251-supplementary.pdf]

Supplementary materials:

Flammulina filiformis Pkac Gene Complementing in *Neurospora crassa* Mutant Reveals Its Function in Mycelial Growth and Abiotic Stress Response

Yayong Yang [1,2], Bin Xie [2], Zhuohan Jing [1,2], Yuanping Lu [2], Jun Ye [1,2], Yizhao Chen [1,2], Fang Liu [2], Shaojie Li [3], Baogui Xie [2,*] and Yongxin Tao [1,2,*]

[1] College of Horticulture, Fujian Agriculture and Forestry University, Fuzhou 350002, China
[2] Mycological Research Center, College of Life Sciences, Fujian Agriculture and Forestry University, Fuzhou 350002, China
[3] State Key Laboratory of Mycology, Institute of Microbiology, Chinese Academy of Sciences, Beijing 100101, China
* Correspondence: fjxiebg@fafu.edu.cn (B.X.); taoyongxin@fafu.edu.cn (Y.T.); Tel.: +86-0591-83789281 (Y.T.)

Table S1. The primers used in this study

Primer	Sequence (5′ to 3′)	Descriptio
FfPkac-F<br>*FfPkac*-R	CTCCATGTCTGTGTTAAAGCG<br>CAACGCAGTCCTTTTTTGAAC	Constructing *FfPkac* complement vector
*NcPkac*3′-F-NotIHF<br>*NcPkac*3′-R-AscI	tggagctccaccgcggtgGCTGAAAGGGCCTCAGGTCGAC<br>taaaacagccgcacGGCGCGCCAACGATTGGCGGGCTGAT	Constructing *FfPkac* complement precursor
*NcPkac*5′-F-AscI<br>*NcPkac*5′-R	gcccgccaatcgttGGCGCGCCGTGCGGCTGTTTTAGGCG<br>taacacagacatggagGTTGGATGCGGACCAGTGAG	Constructing *FfPkac* complement precursor
TrpC-F<br>TrpC-R-SpeIHF	aaaaaaggactgcgttgCCGGGATCCACTTAACGTTACTGAA<br>cgggggatccACTAGGGCGTAGAGGATCCTCTAGAAAGAAG	Constructing *FfPkac* complement precursor
Hyg-F<br>Hyg-R	GACAGAAGATGATATTGAAGGAGC<br>GATTTCAGTAACGTTAAGTGGAT	Hygromycin gene was used for monokaryotation verification
NcPkac-F<br>*NcPkac*-R	CCTGATACCGAGTGCTATCA<br>CGTCCTGTAATAACTCCGTG	*NcPkac* gene was used for monokaryotation verification
SUR-F<br>SUR-R	GCCTTCCGACAAAATGACACC<br>CACGAGCGAGCGAACAAACAG	Chlorimuron-ethyl gene was used for transformation verification
*FfPkac*5′-F<br>*FfPkac*5′-R	CTATTCGCAGTTGTGGTGG<br>TGAATGGTGACTGAATGCC	Validation of the insertion site of the complement vector
*FfPkac*3′-F<br>*FfPkac*3′-R	CTCAGACTCGTTACGGCAA<br>CGTTGTTCCCTCACTTCTTT	Validation of the insertion site of the complement vector
GAPDH-qF<br>*GAPDH*-qR	CCTCTGCTCACTTGAAGGGT<br>GCGTTGGAGATGACTTTGAA	Internal control gene for RT-qPCR
ACTB-qF<br>*ACTB*-qR	GATCGTATGCAGAAGGAGTTGACAC<br>CCACTCTCGTCGTACTCTTGCTTG	Internal control gene for RT-qPCR
Ras-qF<br>*Ras*-qR	TCAATGCGACGAGTAAAGAGAGG<br>CATAGGTCCCACATCTACATTTCG	Internal control gene for RT-qPCR
FfPkac-qF<br>*FfPkac*-qR	ATCCTTCCGCCTCAGAAAGTCATA<br>CGCTGACGGGATTGTGGGC	Detecting expression level of *FfPkac* gene by RT-qPCR
gene1803-qF<br>*gene1803*-qR	TCGCACAGGAAGTCAATACTAACAC<br>TAAGACGTACAAAGATCCAGAAACA	Detecting expression level of *gene1803* gene by RT-qPCR
gene3092-qF<br>*gene3092*-qR	TTCTTGGACCGCAGCAACTTT<br>CATCTCTTCCAGCAACGAGGG	Detecting expression level of *gene3092* gene by RT-qPCR
gene3276-qF<br>*gene3276*-qR	TCAGGACGCCGTCAGAGAGA<br>GATGGGAATCGGAGTGGTGC	Detecting expression level of *gene3276* gene by RT-qPCR
gene3962-qF<br>*gene3962*-qR	AAATGCCAACGCAACAATACG<br>TGACTTTTCTGGAGCGAGAGC	Detecting expression level of *gene3962* gene by RT-qPCR
gene4152-qF<br>*gene4152*-qR	CCGAACTGACGAAGCGTGTC<br>GCGGCGTATTGCTCGAATAA	Detecting expression level of *gene4152* gene by RT-qPCR
gene4386-qF<br>*gene4386*-qR	TGTTGAGGTTCCTTCACTCTTCC<br>GCGTATCCATCTTGTGCGATATA	Detecting expression level of *gene4386* gene by RT-qPCR
gene5702-qF<br>*gene5702*-qR	CTCTATGGACCGCCTATCACG<br>ATCAGCATTGGTGGGGTCTTC	Detecting expression level of *gene5702* gene by RT-qPCR
